# COVID-19 response strategies: considering inequalities between and within countries

**DOI:** 10.1186/s12939-020-01254-9

**Published:** 2020-08-12

**Authors:** Lincoln Leehang Lau, Natalee Hung, Kendall Wilson

**Affiliations:** 1 International Care Ministries Inc, Manila, Philippines; 2 Dalla Lana School of Public Health, University of Toronto, Toronto, Canada; 3 School of Public Health and Health Systems, University of Waterloo, Waterloo, Canada

**Keywords:** COVID-19, LMICs, Pandemic response, Inequalities

## Abstract

Globally, the COVID-19 pandemic has been uncharted territory, and countries and governments have faced the challenge of implementing response strategies to manage local transmission. High-income settings have the resources to devote significant resources to testing, isolation, and contact tracing. Lower-income settings are pressured to emulate such initiatives, but often lack the resources and infrastructure to do so. We highlight the impact of these between-country inequalities, the within-country inequalities, and the potential magnification of unintended consequences due to COVID-19 control measures.

## Background

Since the emergence of the novel coronavirus disease 2019 (COVID-19), policy makers and governments have had to make important decisions around what mitigation and control measures to implement. Given initial uncertainties about the nature of the disease, responses were varied country to country. Over time, three strategies have become recognized as the “backbone” of the response to COVID-19: testing, isolation and contact tracing [1]. In certain countries, these measures appear to have contributed to reductions in the incidence of COVID-19 [2]. Many low- and middle-income countries (LMICs), however, do not have the resources nor the infrastructure to emulate such initiatives to the same extent, and yet may feel pressured to do so. There is a need to consider the inequalities that exist, both *between* countries and *within* countries, and ensure that response strategies are appropriate to the capacity of each jurisdiction.

## Main text

### Between-country inequalities

**Fig. 1:**
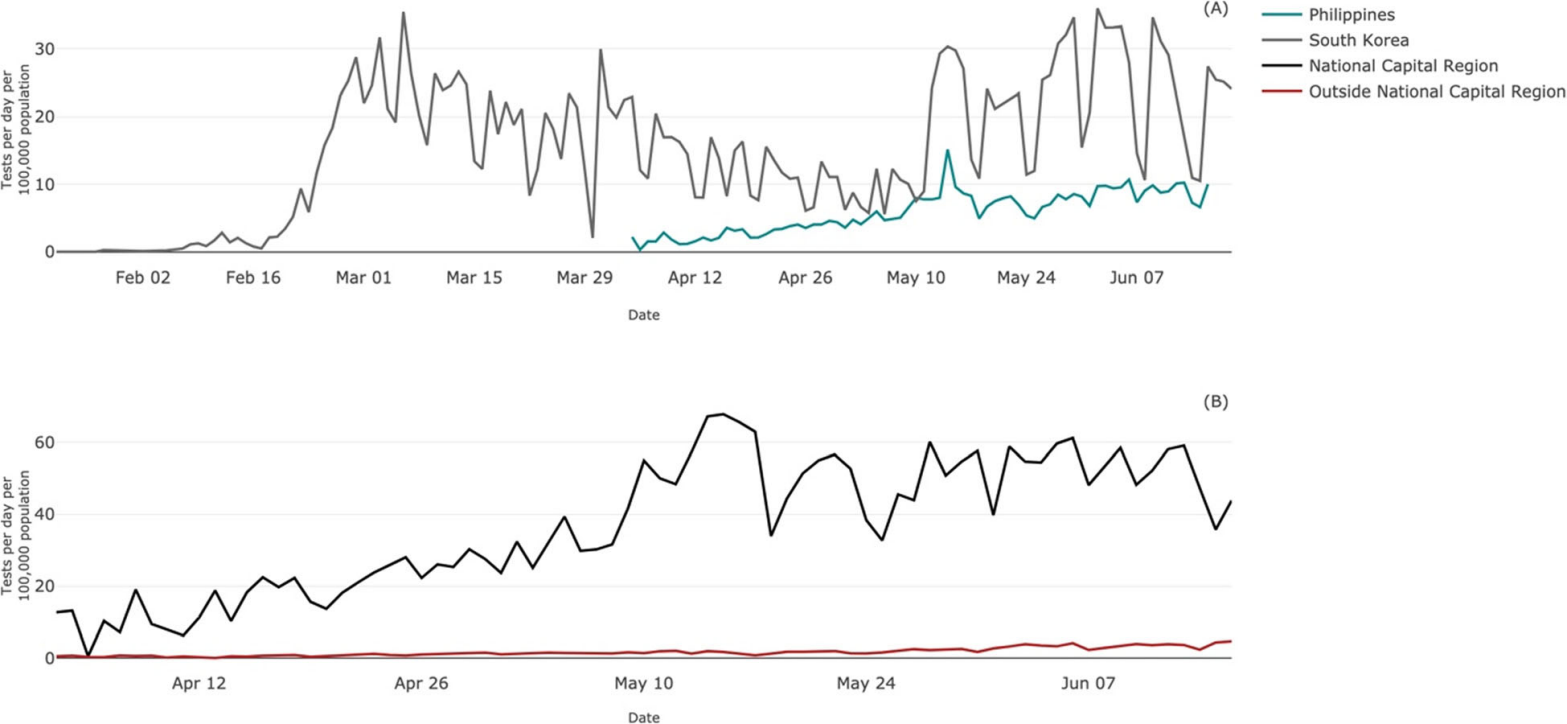
Number of COVID-19 tests conducted per day per 100,000 people in (**a**) the Philippines and South Korea and (**b**) inside the national capital region (NCR) of the Philippines and regions outside the NCR. *Sources:* (A) Reprinted from Max Roser, Hannah Ritchie, Esteban Ortiz-Ospina and Joe Hasell. Coronavirus Pandemic (COVID-19): OurWorldInData.org; 2020, Available from: https://ourworldindata.org/coronavirus accessed June 19, 2020. (B) Department of Health. COVID-19 TRACKER: Republic of Philippines Department of Health; 2020, Available from: https://www.doh.gov.ph/covid19tracker accessed June 19, 2020

These, perhaps unrealistic, pressures have been visible in the Philippines. Initially, the government responded by enforcing strict home quarantines and a lockdown on business and transportation in regions with significant COVID-19 cases. More recently, the focus has turned towards scaling-up national capacities for mass testing, with the expectation that the Philippines should follow in the example of other Asian countries. Researchers in the Philippines have commended Singapore and South Korea’s successful suppression of the reductive number by increased testing and contact tracing [3]. In another report by the University of Philippines, authors made recommendations to expand nationwide testing capabilities to a capacity similar to that of South Korea [4] – but it is difficult to see how this would be feasible in the Philippines. Beyond the procurement of COVID-19 test kits, mass testing would require substantial investment in equipment, lab facilities, and manpower. However, health expenditure per capita in South Korea in 2017 was USD$2283.07, compared to USD$132.90 in the Philippines [5]. South Korea has established almost 600 COVID-19 testing centres [6], and the Philippines currently has 58 [7]. Although the Philippines has been able to gradually expand testing capacity, with only 10 tests conducted per 100,000 population on June 16, 2020, compared to 27 tests per 100,000 population in South Korea on the same day (Fig. 1a), it is evident that the two countries are on different playing fields. The government is seeking to adopt measures that seem to be successful in other countries, but should give due consideration to the constraints and the between-country inequalities that may prevent them from achieving similar outcomes.

### Within-country inequalities

We highlight not only the inequalities between countries, but the disparities that are further magnified when we look within countries and toward regions that have less resources. Local government units (LGUs) have been expected to enforce measures that are consistent with those in the National Capital Region (NCR), but the geographical, social and economic landscape from which these directives originate often differ from the rural communities in which they are meant to be implemented. While 12% of the nation’s population lives within NCR, the region accounts for 36% of the total GDP [8]. Just as the Philippines lacks the resources and infrastructure to match South Korea’s COVID-19 response, regions outside of NCR likewise do not have the resources to replicate the proposed ambition of mass testing in the capital region. Between April 3 and June 16, 2020, 75.5% of all COVID-19 tests were conducted in NCR, and Fig. 1b compares the daily number of tests per 100,000 population that were conducted by location. We note that before regions were assigned COVID-19 labs within a ‘zoning area’ on April 23 [9], all specimens were tested in NCR, even if they originated from individuals elsewhere. However, the gap in testing capacity has since continued to widen, and the difference in number of tests conducted daily per 100,000 population is now almost tenfold.

Another pressing challenge in light of these inequalities are the other health issues that will persist, if not be exacerbated by this public health emergency [10]. The expansion in testing capacity achieved within NCR has been resource intensive, facilitated by public and private sector investment. However, given persisting resource limitations, many tuberculosis (TB) testing facilities have been reconfigured for COVID-19 testing in order to meet demands [11]. These facilities were already limited in regions outside of NCR, but due to resource disparities and the pressure to keep up with the capital region, LGUs have implemented COVID-19 control strategies at the expense of other disease control initiatives. Without proper and timely guidance, many local governments are left to copycat national measures that may be ill-fitting to their communities with resulting social and economic consequences. If the repercussions of ineffective responses could be compounded by unintended harms, it is imperative that due consideration is given to what LGUs are *capable* of doing given local resource constraints.

## Conclusions

In an analysis of the challenges the Philippines has faced raising testing capacity [12], we agree with the authors’ appeals for the integrity of testing to be upheld despite pressures to rapidly scale up. Increasing testing capacity without compromising quality, however, requires technical capacity and significant resources. This raises the question: in regions that do not have the means, what should they do? Given inherent disparities in how resources are distributed, countries should not be treated homogenously when designing national response strategies. While there has been increasing recognition that COVID-19 response strategies need to be context-specific, discussions have primarily been focused on the national level, while differences within the country have tended to be overlooked. The global health community must be sensitive to *both* between and within-country inequalities as formal guidance for low-resource contexts are developed.

National and local governments have been under immense pressure to act and contain the virus, but inappropriately copying approaches could create more unintended harm than good. We have highlighted the observed and anticipated disparities in COVID-19 testing, but other aspects of COVID-19 responses also warrant further exploration. While the fight against COVID-19 is far from over, we need to appreciate between- and within-country inequalities to formulate appropriate, rather than reactionary, pandemic management strategies.

## Data Availability

All data are publicly available through the Our World in Data and the Republic of Philippines Department of Health website.

## Abbreviations

COVID-19: Coronavirus disease 2019
LMICs: Low- and middle-income countries
LGUs: Local government units
NCR: National Capital Region
